# A Personalized Patient Preference Predictor for Substituted Judgments in Healthcare: Technically Feasible and Ethically Desirable

**DOI:** 10.1080/15265161.2023.2296402

**Published:** 2024-01-16

**Authors:** Brian D. Earp, Sebastian Porsdam Mann, Jemima Allen, Sabine Salloch, Vynn Suren, Karin Jongsma, Matthias Braun, Dominic Wilkinson, Walter Sinnott-Armstrong, Annette Rid, David Wendler, Julian Savulescu

**Affiliations:** a University of Oxford; b National University of Singapore; c Yale University and The Hastings Center; d Monash University; e Hannover Medical School; f Independent Researcher; g Julius Center of the University Medical Center Utrecht; h University of Bonn; i John Radcliffe Hospital; j Murdoch Children’s Research Institute; k Duke University; l NIH Clinical Center

**Keywords:** Advance directives, algorithm, generative AI, large language models, Patient Preference Predictor, substituted judgment

## Abstract

When making substituted judgments for incapacitated patients, surrogates often struggle to guess what the patient would want if they had capacity. Surrogates may also agonize over having the (sole) responsibility of making such a determination. To address such concerns, a Patient Preference Predictor (PPP) has been proposed that would use an algorithm to infer the treatment preferences of individual patients from population-level data about the known preferences of people with similar demographic characteristics. However, critics have suggested that even if such a PPP were more accurate, on average, than human surrogates in identifying patient preferences, the proposed algorithm would nevertheless fail to respect the patient’s (former) autonomy since it draws on the ‘wrong’ kind of data: namely, data that are not specific to the individual patient and which therefore may not reflect their actual values, or their reasons for having the preferences they do. Taking such criticisms on board, we here propose a new approach: the *Personalized* Patient Preference Predictor (P4). The P4 is based on recent advances in machine learning, which allow technologies including large language models to be more cheaply and efficiently ‘fine-tuned’ on person-specific data. The P4, unlike the PPP, would be able to infer an individual patient’s preferences from material (e.g., prior treatment decisions) that is in fact specific to them. Thus, we argue, in addition to being potentially more accurate at the individual level than the previously proposed PPP, the predictions of a P4 would also more directly reflect each patient’s own reasons and values. In this article, we review recent discoveries in artificial intelligence research that suggest a P4 is technically feasible, and argue that, if it is developed and appropriately deployed, it should assuage some of the main autonomy-based concerns of critics of the original PPP. We then consider various objections to our proposal and offer some tentative replies.

## INTRODUCTION

*S, a 26-year-old married woman, collapses at home early one morning. When the ambulance arrives, she is found to be in cardiac arrest. She is resuscitated and transferred to hospital but is found to have sustained a severe brain injury from lack of oxygen. S has a long period of hospitalisation and rehabilitation, but six months later has made minimal recovery and is diagnosed as being in a persistent vegetative state. She is dependent on artificial nutrition and hydration. Health professionals discuss with S’s family whether to continue this treatment, or to withdraw it and allow her to die. However, S had made no advance directive, and her husband and parents strongly disagree about what she would have wanted in such a situation.* ^1^

When a formerly competent person like S loses the capacity to make their own medical decisions, others must make such decisions on their behalf. Ideally, the person now lacking capacity (hereafter, “the patient”) would previously have indicated their treatment preferences in an advance directive or similar advance-care planning tool (that is, when they had “full mental capacity”; see Toomey et al. 2023, for a recent discussion). However, in practice, most people do not make use of this option (Silveira 2022). Moreover, even when an advance directive is available, it may often fail to cover situations or treatment dilemmas an individual ends up facing. This can put surrogate decisionmakers, such as family, into a difficult position, as they must decide what to do on behalf of another person whose self-regarding preferences they cannot currently confirm, often in very stressful, high-stakes situations.

Nevertheless, where advance directives are not available, or do not cover the medical situation for which a patient like S requires treatment, current clinical practice relies heavily on surrogate decision-makers to make such treatment decisions. These surrogates may, of course, draw on various factors in deciding what should be done; however, in cultures where individual choice is highly valued, it is typically argued that surrogates should use a "substituted judgment" standard out of respect for the patient’s (former) autonomy: they should choose as the patient themselves would choose if they were currently able to do so (for discussion of the U.S. legal standard, see Dresser 2014).

This is not a matter of universal consensus. Some would argue, for example, that considerations of family well-being should (also) guide substituted decision making, even if this conflicts with what the patient would have wanted (see Box 1 for discussion). However, as Berger (2005) notes, deep conflicts of this kind may not be very common, even in characteristically “individualistic” societies like the United States. This is because a concern for the well-being of one’s family typically is a major factor in one’s own preferences and interests, including those relating to medical treatment. In any case, in this essay, we will focus primarily on situations in which the assumed or attempted goal of surrogate decision-making is to choose the course of action the patient would endorse if currently autonomous (i.e., without thereby taking a stand on whether this goal should always be favored over others).

Even if such a goal is assumed, however, there are obstacles to taking this approach. One is that human surrogates are often mistaken about what a patient would have preferred, despite making a good-faith effort to adhere to the prevailing substituted judgment standard. Even under ideal research conditions, when it is unclear which treatment is clinically indicated, surrogates’ probability of correctly identifying what treatment an individual would want if competent is only slightly greater than chance (Ciroldi et al. 2007; Shalowitz, Garrett-Mayer, and Wendler 2006; Stocking et al. 2006). Another obstacle, as mentioned, is that surrogate decision-makers often experience considerable anxiety and distress in response to being asked to make, and feeling responsible for, what are in many cases literal life-or-death decisions for their loved ones (Jongsma and van de Vathorst 2015; Rid and Wendler 2014a).

In response to such challenges, David Wendler, Annette Rid, and colleagues have called for the development of a “Patient Preference Predictor” (PPP): a computer-based algorithm that could be used to supplement (or in some versions of their proposal, possibly replace) key aspects of a typical surrogate decision-making process (Shalowitz, Garrett-Mayer, and Wendler 2007; Rid and Wendler 2014a; Rid and Wendler 2014b; Wendler et al. 2016; see also Ditto and Clark 2014). The PPP would be based on a large, voluntary survey of the general population in which participants would be asked to provide their preferences in various hypothetical medical situations alongside demographic data, such as age, gender, and insurance status. These demographic data would then be correlated with the expressed medical preferences of the surveyed participants, who would ideally be randomly drawn from, and representative of, the target population (Rid and Wendler 2014a). These correlations, in turn, would be used to construct an algorithm for inferring the preferences of particular persons who have become incapacitated and require medical care (i.e., including those whose preferences were *not* part of the original data set as they were not among the surveyed participants).^2^

This proposal is based on a robust empirical relationship that has been demonstrated in numerous studies: namely, that demographic variables such as the ones just mentioned are statistically predictive, in the aggregate, of people’s healthcare choices as elicited under various conditions (reviewed in Rid and Wendler 2014b). So, for instance, a PPP based on such data might find that most young women of a similar age and background to S—the comatose patient from our opening example—would not wish for continued tube feeding under such-and-so conditions. To the extent that (a) those conditions apply to S’s case, (b) S is relevantly like other members of her (multiply intersecting) demographic categories, and (c) such information could meaningfully be measured or quantified to a reasonable approximation and entered into a PPP, a prediction might then be made as to what S herself would want in the current circumstances (within a certain level of confidence). Indeed, available findings suggest that a PPP’s predictions of patient treatment preferences might well be more accurate than those of most next-of-kin human surrogates (Smucker et al. 2000; Houts et al. 2002; Shalowitz, Garrett-Mayer, and Wendler 2007).

In short, the PPP is intended to help surrogates, in a shared decision-making process with clinicians, make treatment decisions that are more likely to be consistent with the patient’s own preferences (provided, again, that these are not too idiosyncratic as compared to those of otherwise similarly situated persons of a relevantly similar background).

Nevertheless, the very idea of a PPP has been met with various objections. Some argue that respecting patient autonomy requires more than just accurately predicting treatment preferences based on population data (John 2018; Sharadin 2018). On a strong formulation of this view, medical decisions made on behalf of formerly autonomous persons should incorporate *only* the reasons, values, and evidence that the person themselves took, or would have taken, into account when making treatment decisions (John 2014; John 2018).^3^ Other concerns include that patients may have preferences about how treatment decisions are made for them, including how their preferences are identified, not just which treatments they receive (Mainz 2023). In other words, it may be that substituted decision-making should consider not only patients’ *treatment* preferences, but also their *process* preferences (i.e., the process by which treatment decisions are made or by which their treatment preferences are inferred). Only then could a patient’s autonomy robustly be respected.

In this paper, we will look at several such concerns regarding the PPP, rejecting some, but accommodating others. Our main purpose, however, will be to argue that at least some of what we take to be the most forceful objections to the PPP could in principle, and likely also in practice, be assuaged by a novel adaptation of the original idea. Here, we introduce and defend the notion of a *Personalized* Patient Preference Predictor (P4).^4^

We propose to use machine learning to extract patients’ values or preferences from individual-level material produced primarily by themselves in which their preferences are likely to be encoded (if only implicitly). This hypothetical model for predicting patient preferences would harness advances in generative artificial intelligence (AI) to create large language models (LLMs) adapted to (that is, fine-tuned on) a person-specific corpus of text (as in Porsdam Mann et al. 2023). The result would be a kind of ‘digital psychological twin’ of the person (roughly along the lines discussed in de Kerckhove 2021) that could be queried in real-time as to the patient’s most likely preferences for treatment in any given healthcare crisis.^5^ In short, the P4 would be a personalized, rather than population-based, patient preference predictor.

In principle, a wide range of person-specific fine-tuning material could be used for model-training purposes*, assuming* that all relevant permissions and data protection measures were in place (a delicate matter to which we return below). Some current possibilities for such material are summarized in Table 1. Applied to the case of S, we might find that a P4 trained on such material could indicate—based on her own prior writing and other digitally recorded behavior—that there is a high chance she, specifically, *would* wish for artificial nutrition and hydration to be continued (even if that is not what most women of a similar age, etc., would choose in such a situation).

**Table 1.** Different versions of the proposed P4 based on different information types as input.

1) A P4 trained on writing produced directly by an individual, such as emails, blog posts, or social media posts. Such text might then be supplemented by additional digital information reflecting the individual’s past choices or behavior, such as treatment decisions encoded in electronic health records (or even Facebook ‘liking’ activity; see Lamanna and Byrne 2018). For technical reasons, such information would need to be stored as writing; however, one important way in which such information could be obtained would be through advances in speech-to-text transcription software. For example, physicians might, with permission, record and automatically transcribe conversations with individual patients. 2) An enhanced P4 trained, instead or in addition, on explicit responses provided by an individual, while competent, to questions relating to their hypothetical treatment preferences under various conditions (i.e., an individual-level version of the population-level surveys proposed for the original PPP). This could take the form of questionnaires or interviews with healthcare providers, perhaps as part of a regular checkup, while waiting for care, or in the context of more structured advance care planning (for a detailed proposal as to how this might be done in practice, see Ferrario, Gloeckler, and Biller-Andorno 2023a). 3) Perhaps more ambitiously, and to get at underlying values or preferences that might not be consciously accessible to most people (i.e., for purposes of self-report), individuals could be incentivized to participate in specially designed, value-eliciting discrete choice experiments (see, e.g., Ryan 2004) in which they would need to decide between options in a sequence of tradeoffs pitting various decision-relevant factors against one another. These could potentially be ‘gamified’ and delivered by way of a downloadable mobile app, an appropriately secured computer interface in healthcare waiting rooms, a publicly accessible internet-based platform associated with user accounts, etc. For a technical description of how preferences elicited in this, or a similar manner, might be integrated with other information (e.g., medical data) in a shared decision-making context, see the work by Sacchi et al. (2015). 4) A P4 trained on the above types of information, if available and appropriately authorized, but if not (or in addition), on responses to questions concerning a patient’s likely or known medical preferences made by surrogate decision-makers and other persons close to the patient. Most likely, such data would be collected after a patient loses capacity, with the responses from surrogates integrated and weighed according to the parameters of the algorithm (i.e., for purposes of predicting what the patient would choose in the particular situation that has arisen). 5) A P4 fine-tuned on any of the above-mentioned datasets, but whose base model is not a generic LLM but one trained on population-level data, whether responses from large-scale surveys as in the original PPP proposal (Rid and Wendler 2014a), or population-level electronic health record data linked to social media activity, as per the suggestion of Lamanna and Byrne (2018).

In this paper, we discuss how the P4 would work and how it compares to the PPP. In particular, we discuss how the P4 might improve upon the PPP, both in terms of predictive accuracy and respect for autonomy. We conclude that the P4 likely would be superior to the PPP in both respects; however, we do not suggest the two proposals are mutually exclusive (for example, a PPP could be used when sufficient data or time to develop a P4 are not available). In any case, given the importance of improving treatment decisions for incapacitated persons in various time-sensitive healthcare situations, we suggest that the development of such preference predictors, both technically and in terms of crafting associated ethical guardrails, should urgently be pursued with the involvement of all relevant stakeholders. These include ethicists, healthcare professionals, AI experts, patients, and members of the general public.

We begin by outlining the concept of the P4 at a high level. We then turn to thorny questions of implementation, including the role of the P4 in shared decision-making and issues of privacy and consent for the use of training data. Finally, we explore the extent to which our P4 proposal can meet a number of objections levied against the PPP.

## THE P4

The P4 is a hypothetical personalized version of the PPP. It is personalized in the sense that it relies on information produced by, describing, or otherwise pertaining to a specific individual, beyond mere demographic categorization. Depending on the type and variety of information that would be available and duly authorized for model training purposes, a P4 could take one of a number of forms, either alone or by combining features from different versions, as summarized in Table 1.

Here is how we envision the P4 would work. Ideally with the patient’s prior permission, but if not, with their surrogate’s permission, it would draw on various types of personal data as described in Table 1 in order to predict (a) their first-order treatment preferences during periods of decisional incapacity, (b) their second-order preferences for how treatment decisions are made for them during these periods (e.g., with respect to the type or degree of desired family involvement, assuming that such preferences were not explicitly recorded in an advance decision-making instrument), and (c) how *certain* the patient is about these preferences and how strong the preferences are: for example, are their preferences regarding which treatments they receive stronger or less strong than their preferences regarding family involvement?

Specifically, the P4 would take the form of a fine-tuned LLM trained on text produced by, or describing, an individual. It is this aspect of the P4 that distinguishes it from related proposals, such as the brief sketch of a similar idea mentioned by Biller-Andorno and Biller in 2019.^6^ Similarly, Lamanna and Byrne (2018) have proposed the use of an ‘autonomy algorithm’ “to estimate confidence for predicted preferences of incapacitated patients by using machine learning technologies to analyze population-wide data sets, including EHRs [electronic health records] and social media profiles” (907). Our proposal differs from that of the latter authors in specifying the use of fine-tuned LLMs for this purpose, as well as in focusing primarily on individual-level patient information rather than “population-wide datasets” for model training.^7^

*Fine-tuning* is a process in which the last few layers of an LLM’s neural network are exposed to a specialized corpus of text, such that the resulting model retains its fundamental representation of language but generates output that is influenced by the more specific features of the specialized training set to which it was exposed (Church, Chen, and Ma 2021). A P4 as here envisioned would use, for its specialized training set, text written by or describing an individual. Ideally, this text would be medically relevant: for example, as mentioned, data stored in electronic health records and biobanks; responses to medical questionnaires or value-eliciting choice experiments undertaken by the individual while competent; and so on.^8^

In addition to these primary, health-related sources, other text produced by individuals, such as blog posts or other published writings (Porsdam Mann et al. 2023), social media activity (e.g., Facebook ‘likes’; see Lamanna and Byrne 2018), and emails could be used; as could purchasing and browsing histories (see below for consent and privacy concerns regarding the use of such materials). To maximize effectiveness, as much of this information as can safely and ethically be gathered would be used as input to the P4 fine-tuning process. However, where an individual has previously indicated that some information should not be used, for example social media posts from more than a decade ago which no longer reflect their current preferences, values, or personality, such requests should be honored.

In general, our description of the P4 is premised on the idea that individual preferences, including not only first-order treatment preferences but also second-order “process” preferences for how treatment preferences are inferred, can be elicited directly or indirectly from such data. Of course, much empirical work will be needed to establish the extent to which these or other data sources can in fact allow accurate inferences of an individual’s first- or second-order medical preferences; in future papers, we hope to sketch out such an empirical research program. However, compared to the current baseline accuracy of human surrogate decision-making, which seems to hover around chance, a P4 would only have to be somewhat more accurate than that to be useful for present purposes. Based on current trends, this seems more than plausible.

It should also be noted that it is currently possible to manipulate the weight that an LLM—such as the proposed P4—places on potentially more relevant information, such as questionnaire data or information stored in electronic health records, during the fine-tuning process. One technically simple way in which this could be done would be to specify a greater number of training epochs (i.e., the number of times the underlying model is exposed to the fine-tuning data) for a privileged, medically-relevant dataset, and a lower number of training epochs for perhaps more tangentially relevant information such as purchasing history. Any such adjustments should be explored during the development of our proposed P4 and evaluated with respect to their impacts on accuracy.

Although such a specialized system has not, to our knowledge, yet been developed or tested, it is inspired by, and is technically analogous to, existing fine-tuned LLMs for which we do have a better sense of functionality. For example, in a recent proof-of-principle exercise, an LLM fine-tuned on the previously published writings of three of the present authors appeared to adapt to the style, argumentation, and reasons employed in those individuals’ prior writings (Porsdam Mann et al. 2023). Another LLM fine-tuned on philosopher Daniel Dennett’s writings has produced outputs convincingly similar to Dennett’s own responses to novel questions not addressed in the model’s training set (Schwitzgebel, Schwitzgebel, and Strasser 2023).

These early studies concern the adaptation of LLMs to individuals’ styles of writing and argumentation, rather than to their medical treatment preferences *per se*. Indeed, outside of a small number of informal experiments we have been engaged in ourselves, we are not aware of any existing work that has attempted to infer such treatment preferences using LLMs. However, recent work has shown that LLMs are currently able to infer non-medical preferences to a high degree of accuracy across a variety of contexts, often using only limited examples of relevant conduct or information. For example, they can infer movement preferences for individualizing robotic systems (Wu et al. 2023); they can predict individuals’ movie ratings, reflecting their preferences for certain types of cinema (Kang et al. 2023); and they can anticipate individual responses to survey questions based on one’s answers to other surveys (Kim and Lee 2023).

Although it is no small leap from, say, predicting film ratings to predicting life-or-death decisions about health, there are good reasons to think that an appropriately fine-tuned LLM would be able to infer medical preferences. In addition to the above-mentioned studies showing that LLMs can infer non-medical preferences, there is a large literature on the related notion of aligning AI systems such as LLMs with human values and preferences: both in general (Askell et al. 2021; Gabriel 2020; Christian 2020; Kenton et al. 2021), and for specific individuals (Kirk et al. 2023). In either case, the aim of research is to identify a process that can successfully adapt LLMs to reflect human values and preferences.

Finally, recent work has shown that LLMs can outperform humans in the creation of consensus statements based on homogenous preferences expressed in written opinions (Bakker et al. 2022). This also goes to show the extent to which LLMs can infer preferences based on textual inputs. In general, the primary function of LLMs is prediction: given data of a sufficient quality and relevance, *prima facie* LLMs should be able to predict medical preferences, too. This is a key assumption for our purposes, and it bears repeating that future work is necessary to confirm its validity, especially in the context of health.

## IMPLEMENTATION, PRIVACY, AND CONSENT

There are various potential approaches to implementing a P4 in practice, each with different implications for ethical concerns such as privacy and consent (see Senthilnathan and Sinnott-Armstrong, forthcoming, for details).

At least initially, the P4 should be implemented in a manner consistent with currently existing legal and ethical norms governing healthcare decision-making for incapacitated persons in a given jurisdiction. Where surrogates such as family members have legal authority, they should determine the appropriate role and use of a P4 in the decision-making process. Ethically, too, this may be valuable insofar as some patients not only care about predictive accuracy but also about the process of decision-making and want their family members to be involved (see Box 1). Elsewhere, patients should be asked prospectively to indicate the extent to which, and manner in which, they would like a P4 to be used in case of decisional incapacity.

Whenever possible, the use of a P4 should be voluntary: individuals should be asked about how, if at all, they would like a P4 to be used in case they lose decisional capacity, about the types of information, if any, that should go into it, about the priority it should be given (e.g., the weight that should be assigned to its predictions versus those of human surrogates), and so on. While we expect that our proposed P4 would be a help, rather than a hindrance, for substituted decision-makers, making its use voluntary for surrogates (if there are no prior binding instructions from the patient to the contrary) would also address any worries related to possible conflicts between the preferences or decisions of human surrogates and the predictions of a P4.

Searching a patient’s personal communications and medical records might raise fears that using such data to construct a P4 will violate the patient’s rights to privacy. However, the patient’s rights to privacy include the right to share data if and as they want, so they can consent to this use of their data. In addition, the data on which the P4 is based could be stored securely so that this information cannot be accessed for purposes other than constructing a P4 without the person’s consent.

Similar concerns about uploading such private information for the purposes of fine-tuning LLMs could be obviated through the use of locally stored LLMs: While current state-of-the-art models are operated by private, for-profit companies, there are also many alternatives (examples at the time of writing include GPT4All, Orca Mini-GPTQ, and LLaMA 2 Chat-GPTQ, as well as many others) which can be downloaded and hosted on local computers, thus removing the need to upload potentially sensitive information. A third alternative would be the use of a privately or publicly operated service specifically designed for the purpose of delivering a P4-as-a-service; such a company or public service could then be designed with privacy protection in mind.

Of course, the use of a PPP or P4 is itself predicated on the absence of sufficient advance information to determine first-order treatment preferences directly. Predictive algorithms in fact offer the most benefit in cases where there are no other feasible options for determining a patient’s preferences (e.g., human surrogates are not available) as argued by Jardas, Wasserman, and Wendler (2022). This may be a large proportion of cases of patients who lack capacity. Despite concerted efforts to improve uptake, too few patients have completed an advance directive (Wendler et al. 2016). Even among those who have, there are often difficulties in documenting treatment preferences without adequate counseling due to both missing or mistaken knowledge about future medical possibilities and their concrete implications (Dresser 2014),^9^ and the high psychological burden of making certain decisions (e.g., about one’s own end-of-life care). A P4 may thus help to improve advance care planning.

In cases where individuals have not consented in advance to the use of a P4, various options are available. One is to proceed based on proxy consent given by surrogate decision-makers or next-of-kin. Another is to use all *publicly* available information about the individual patient that is not constrained by data protection laws. A third is to proceed irrespective of the lack of explicit prior consent. Finally, a fourth option is not to proceed without such consent. We do not here wish to take a position on this difficult question, except to say, again, that at least initially these implementation decisions should be made in a manner that respects the ethical and legal standards already in place in the relevant jurisdiction. Later, these standards might then be changed in light of practical experience and academic debate.

Box 1 The role of family.

As in the case of the PPP, some may argue that family members alone, rather than an algorithm such as the proposed P4, should be relied upon to indicate what should be done in situations when their loved one lacks capacity. This may either be out of a belief that family members have an independent claim over the patient’s treatment decisions (a belief that is contrary to the legal situation in many jurisdictions) or out of respect for the patient’s wishes that their family be involved in any surrogate decision-making process (Brock 2014). The level of expected involvement of family members is also likely to vary between cultures.

According to Jardas, Wasserman, and Wendler (2022), the first justification would constitute a radical revision of the current practice in many jurisdictions to respect the independent wishes of the patient, while the second could be addressed by incorporating a PPP (or by the same token, a P4) in such a way as to supplement, rather than supplant, human family-based surrogate decision-making (see also Biller-Andorno et al. 2022; Ferrario, Gloeckler, and Biller-Andorno 2023b; however, for a contrary argument that AI-based patient predictors *should* supplant family as decision-makers if more accurate and less biased, see Hubbard and Greenblum 2020).

Involving family members as surrogate decision-makers for incapacitated patients is often based on the belief that relatives know the patient’s treatment preferences and can therefore best predict what the patient would have wanted (Lindemann and Nelson 2014; Jardas, Wasserman, and Wendler 2022). However, as noted previously, this is not well-founded. In fact, only a small proportion of patients (21.8%) continue to prioritize family involvement when informed that surrogates often do not know the patient’s preferences (Jardas, Wasserman, and Wendler 2022). Thus, it may be preferable to use a system such as a P4—again, at least as a supplement or additional source of information—to reduce the burden of “total” responsibility on family members and minimize considerable anxiety and stress (Jongsma and van de Vathorst 2015; Rid and Wendler 2014a).

While there is no evidence yet about patients’ preferences for the use of a P4 instead of, or in addition to, human surrogates such as family (we are in the process of gathering it), we know that some patients do prioritize minimizing the decision-making burden on their families, in which case the use of a P4 may respect the patient’s process preferences as well. For further discussion, see Bleher and Braun (2022) on “distributed” responsibility for healthcare decision-making (i.e., between humans and AI); see also Allen et al. (2023) on “delegating” certain healthcare practices to an LLM.

## ADVANTAGES OF THE P4

The PPP and P4 are not mutually exclusive. Nevertheless, there are three key advantages to using a P4 as compared to using (only) the PPP. One is that its predictions of first-order patient treatment preferences are likely to be more accurate than those of the PPP. This assumption is based on the relative success of fine-tuned LLMs over general LLMs in inferring non-medical preferences at the individual level as described above. Moreover, the P4 is trained on person-specific information, whereas the PPP uses only group-level data. Thus, the PPP is only able to make accurate predictions at the individual level if the individual’s preferences are sufficiently close to group averages (i.e., they are not a statistical outlier). By contrast, since the P4 is already personalized, there is no such requirement for the P4. In other words, assuming there is enough individual-specific information available to train a P4, there is no further requirement that the individual in question be at all similar to others of a similar background in order for the P4’s predictions to be personally valid.

A second advantage of the P4 compared to the PPP is that the P4 would, potentially, be able to predict a broader range of preferences than the PPP. While the PPP is limited to, or at least likely to be most accurate on, predictions concerning the specific cases included in the surveys on which it is based, the P4 is not necessarily restricted in such a way. This is because LLMs—such as the P4—operate at a different level of generality or abstraction than do algorithms like the PPP. Whereas the latter correlates demographic information to hypothetical choices regarding specific, pre-determined medical situations (and is thus useful primarily for those situations), a P4 is more likely to infer the underlying structure of an individual’s preferences as applied across a range of situations, as it draws on the full diversity of sentiments and reasons expressed in one’s corpus of text more generally.

A third advantage of the P4, which gets to the heart our thesis, is that it is less vulnerable to certain types of autonomy-based objections, such as those that have been leveled against the PPP (Wasserman and Wendler 2023). By this statement, we do not mean to imply that we simply accept all such objections as applied to the PPP. Rather, we argue that, whatever one thinks of the force of these objections in relation to the PPP, they have less force, if any force at all, against the proposed P4. We make a case for this view in the following sections.

## AUTONOMY-BASED OBJECTIONS TO PATIENT PREFERENCE PREDICTION

One way of respecting a person’s autonomy in making a substituted judgment is simply to “get the right answer”—that is, to choose what they would choose in the current situation. This standard sets aside the question of means, focusing only on the end achieved. An alternative view, advanced by critics of the PPP, is that the means matter. One version of this objection is that the prediction of an individual’s preferences must be based on information that the individual in question would regard as being appropriately related to the shaping of their own personal preferences, or that they would at least not reject as inappropriate (O’Neil 2022). By this standard, too, the P4, based as it is on individual-specific information, seems to fare better than the PPP, the latter of which relies on broad demographic variables to predict patients’ preferences.

However, critics of the PPP have raised another objection which may be relevant to the P4. According to these authors, the PPP (and presumably also the P4) fails to respect an incapacitated individual’s autonomy because, as a machine, it cannot *appreciate* the reasons and values that underpin patients’ preferences (John 2018; Sharadin 2018). Abstracting away from potential quibbles about what it means to “appreciate” a reason, and whether LLMs should really be excluded from the set of entities that have this (or a relevantly similar) capacity, we think this objection is in danger of proving too much. For it would imply that the P4 is being held to a higher ethical standard than is currently applied to human surrogates.

To see this, let us suppose that a human surrogate correctly states, based on a recent discussion, what a patient would want, but without necessarily knowing, much less fully “appreciating,” the reasons for that preference. If we now suppose that the surrogate’s prediction is used to make, or help make, a substituted judgment for the patient, it seems unlikely that this would trigger concerns about a failure to respect the patient’s (former) autonomy. Instead, we expect that it would be seen as sufficient that the surrogate was likely enough to get the right answer, and to do so having drawn on person-specific data (e.g., a story told to them by the patient) that plausibly captures or otherwise serves to convey the patient’s actual preferences.

This is not to dismiss concerns regarding “black box” algorithms (e.g., Benzinger et al. 2023), which have rightly prompted research into explainable AI systems. However, we should be wary of a potential double standard whereby a P4 would be required to explain its predictions to a greater level of detail than a similarly situated human surrogate. In fact, such a standard may be unrealistic in either case, given that patients’ preferences are not always based on explicit or articulable reasons in the first place. Indeed, as Biller-Andorno and Biller (2019) point out, many patients find it difficult to explain their own preferences when directly asked by clinicians. Given this, it may be problematic to expect that either a P4 or a human surrogate should have to “appreciate” the reasons or values behind a patient’s preferences to respect their autonomy.

An additional autonomy-based objection has been raised by Mainz (2023). Although originally formulated in relation to the PPP, it might also apply to a P4. It is the ‘objection from higher-order preferences’ (alluded to earlier). In this objection, Mainz argues that many people have strong ‘second-order’ preferences about how their preferences are predicted (Mainz 2023; see also O’Neil 2022). For example, just as a patient might have a higher-order preference that their treatment preference not be inferred by means of an algorithm that, in some sense, ‘reduces’ them to their demographic categories (as with the PPP), a patient might likewise have a preference that their treatment preference not be inferred from, say, their social media posts (a possible source of data for the proposed P4).

If individuals do have these concerns, one way to allay them might be to give individuals the option of providing additional information, corresponding to versions 2 or 3 of the proposal in Table 1. A person could provide this information, for example, by filling out a survey that asks general questions about how much certain values matter to them or by reporting decisions in a sample of concrete scenarios (see, e.g., Ferrario, Gloeckler, and Biller-Andorno 2023a) or discrete choice experiments (e.g., van Kinschot et al. 2021). Where such prospective data-gathering is no longer possible because of mobility or capacity issues, a similar process could be carried out instead by persons close to the patient, such as their friends and family. This would correspond to version 4 of the proposed P4 in Table 1.

In addition, policies about how to construct and implement a P4 could incorporate a patient’s preferences for using only certain sources of information. Moreover, the extent to which a P4 would be able to accurately infer second-order preferences from contextual information in its training data is an open question. However, even if it should turn out that a P4 is not capable of making such inferences, this objection can be met by offering the use of a P4 prospectively as a voluntary choice instead of a requirement, such that those who have strong second-order desires about how their preferences are predicted are able to decline the use of a P4. This would make the use or otherwise of a P4 an instance of self-determination, an aspect of autonomy which might be valued greatly by some patients.

## PRACTICAL AND EPISTEMIC LIMITATIONS OF THE P4

We will now consider some practical and epistemic limitations. One concerns how we would evaluate the accuracy of a P4 (or indeed that of surrogates or the PPP). Put simply, it will never be possible to know with certainty what an incapacitated patient would have autonomously preferred in a specific situation, because by definition they are not able adequately to express this preference. The closest we can get to such knowledge is the use of hypothetical scenarios answered by the patient in question prior to incapacity; yet hypothetical situations do not necessarily reflect what people choose in actual situations, amongst other reasons because of social desirability bias in these types of research methods. However, this is a general problem for any kind of prediction, whether based on an algorithm such as the PPP or P4, on a surrogate’s decision-making, or indeed even an advance directive.^10^

Given this problem, it may be that surrogate decision-making has already reached the upper limits of accurately predicting the treatment preferences of incapacitated patients (Kim 2014). While this may be true, the only way to approximate a proof of this thesis would be to launch a full-scale prototype of a PPP (or P4) and compare its accuracy to that of current standards (Jardas, Wasserman, and Wendler 2022). That being said, to be useful, a P4 does not have to predict with absolute certainty the treatment preferences of an incapacitated patient. Some authors hold that it simply must be better than the (barely better than chance) predictive capabilities of human surrogates and in this or other ways help surrogates with their difficult task (Shalowitz, Garrett-Mayer, and Wendler 2006).^11^

Others may argue that being able to predict one’s preferences requires substantial knowledge of the patient’s identity (Tooming and Miyazono 2023). Such knowledge of the self cannot be reproduced on the basis of description alone but requires an in-depth contextual understanding of the patient’s life and individual clinical circumstances. As a form of AI, a P4 would only be able to make empirical inferences based on previously produced textual evidence. Family members, on the other hand, make qualitatively different sorts of inferences based on their capacity to share in the patient’s intentions (Tomasello et al. 2005). However, these two approaches are not mutually exclusive. For example, as mentioned above, it is possible to imagine a variation on a P4 in which family members or others close to an individual are asked to provide relevant information about a patient’s preferences based on their knowledge of a patient’s intentions.

There may also be problems with the implementation of a P4 in clinical practice, similar to the difficulties that already exist with advance directives. For example, the latter are often not available when needed or not followed by healthcare professionals. It seems likely that these same problems would apply to attempts to have individuals fill out various surveys in advance designed to elicit their preferences and values (i.e., to be able to feed these particularly rich, personalized datasets into a P4 to further improve its inferences). We do not deny that there would be various barriers to uptake. Nevertheless, where such advance information could in fact be provided for the purpose of training a P4, it would likely provide numerous benefits in terms of the accuracy of prediction due to a closer fit between the training data and actual preferences, and thus the strength and directness of the inferential connection between the two. And even where such personalized survey data could not be collected in advance, it is possible that other potential sources of individual-level data that might be easier to collect (e.g., social media posts or other publicly available writing) could still be used to reasonably good effect.

Finally, it is possible that a P4 might be given undue weight in some circumstances. LLMs in general are capable of producing texts that indicate a high degree of confidence. Moreover, the degree of confidence with which LLMs present text is independent of the degree of certainty that the text does indeed correspond to the target construct—in this case, individual preferences or values. There is a chance that highly confident-sounding statements of preferences by a P4 could lead to inappropriate reliance on its output due to an inability to determine the degree to which such statements are based on plausible inferences from training data. This is an important point that needs to be addressed before clinical use of P4s is considered. Methods of addressing it could include technical work aimed at allowing a P4 to compute and express its degree of confidence. Indicating how uncertain the prediction is might reduce over-reliance and would also provide a more realistic representation of the preferences of patients who are themselves uncertain about what to do in difficult cases. These confidence intervals along with additional information concerning the functioning, strengths, and weaknesses of LLMs in general and P4s in particular, could be provided to surrogates and clinical decision-makers.

## CONCLUSION

To return to the case at the start of this paper, how can we improve decision-making for patients like S who have lost capacity and for whom there is uncertainty or disagreement about what they would have chosen? When first introduced, the PPP was conceived as a promising approach to improving treatment decision-making for incapacitated patients. In this paper, we propose a novel means of patient preference prediction using machine learning algorithms, specifically fine-tuned LLMs. We refer to this hypothetical predictive system as the Personalized Patient Preference Predictor (P4). By fine-tuning an LLM on person-specific textual evidence, a P4 may be more accurate at predicting a patient’s actual treatment preferences than the current practice of using surrogate decision-makers. It also may be more accurate than a PPP as well as less liable to certain objections based on respect for individual autonomy.

As our proposal is theoretical only, and as we (or others to our knowledge) have not yet conducted formal studies to empirically test a P4, several things need to happen before it can be stated with confidence that the P4 is, or would be, a superior method of predicting patient preferences. These four points require further consideration but may provide an initial framework for thinking about the P4 as an aid to proxy decision making.

First and foremost, prototype P4s need to be built and their accuracy compared to PPPs and human surrogates. Such prototypes should, at first, include state-of-the-art models fine-tuned only on individual data that is publicly available or for use of which informed consent has been explicitly obtained. This is an important first step for addressing feasibility and privacy concerns. However, prototype P4s using alternative LLMs capable of being stored locally (thus obviating privacy concerns) need also to be explored either in parallel or once feasibility has been established. Likewise, the accuracy of P4s across languages other than those for which LLMs are best suited (English, Chinese, and Spanish) needs to be tested and if necessary language-specific improvements should be pursued.

Secondly, research on P4 prototypes should attempt to establish the relative usefulness of various types of information (e.g., directly medically relevant information gleaned from electronic health records versus more indirectly relevant information such as social media posts) for the purposes of medical preference prediction. This would provide useful information regarding the extent to which P4s should privilege certain types of information during the fine-tuning process (e.g., by weighting these types of information more heavily).

Thirdly, surveys should be carried out in which individuals are asked about their opinions and concerns relating to P4s, as well as whether they would wish to have them used in a hypothetical future in which these individuals become incapacitated and relevant advance directives are not available. Better yet, experimental methods drawn from the cognitive sciences could be used to probe public attitudes and intuitions while systematically varying and testing candidate factors that might be expected to play a role in shaping their perspectives on these questions (Earp et al. 2020, 2021, 2022; Lewis, Demaree-Cotton, and Earp 2023).

Fourthly, more thought should be given as to the ways in which a P4 would be integrated into established advance care planning and surrogate decision-making procedures. Open questions include the potential for mixed-modality preference prediction in which surrogates, PPPs, and P4s are used in combination; as well as the extent to which P4s should be used to supplement or supplant surrogate decision-making in cases of conflict.

Fifth, there is a need to establish evaluation strategies. A central finding of our analysis is that certain structural conditions must be met in clinical decision-making for a P4 to be understood as strengthening patients’ right to self-determination. Embedding the application of P4 in shared decision-making structures is likely to be helpful here. This is especially the case if there are clear protocols that specify the process of using P4 in concrete decision making as well as how to deal with interpretive uncertainties in the assessment of indicated preferences.

Perhaps the most important advance would be a situation in which the P4 could in fact cite the *reasons* for its predictions—that is, at minimum, identifying which statements or behavior it is basing its prediction on. If the P4 is made explainable, either by advances in the underlying LLM itself or by pairing it with higher order explanations through programs such as LIME, it could also in principle infer the weights that the patient places on various considerations and how those considerations interact. This would allow independent scrutiny by health professionals, surrogates, and family. In other words, while it might be difficult or impossible in some cases for LLMs to make their predictions fully explainable, it seems in principle that it would be possible to extract, or zero in on, the relevant factors upon which the predictions are based. These data could then be evaluated by humans to try to determine their meaning (for discussion, see Gloeckler, Ferrario, and Biller-Andorno et al. 2022).

There are wider implications to our discussion. Some of us have proposed the use of AI to enhance not merely prudential decision-making in relation to one’s context-dependent preferences (the subject of this paper) but also moral decision making more broadly (Savulescu and Maslen 2015; Giubilini and Savulescu 2018; Sinnott-Armstrong and Skorburg 2021; Demaree-Cotton, Earp, and Savulescu 2022). ‘Moral AI’ could extract a person’s values from their explicit input, behavior and statements, and retrieve ‘big data’ about options which would best serve those values. AI, in the form of LLMs, could make implicit values explicit, and potentially challenge a person’s moral or prudential values, leading to moral or personal development. In this paper, we have merely touched the tip of the iceberg of the use of AI to engage with a person’s values—the possibilities are enormous and extend well beyond the decision to limit or extend life-sustaining medical treatment.

## Notes

1 This case is loosely based on the case of Terri Schiavo (see Perry, Churchill, and Kirshner 2005). In that case, part of the legal and ethical dispute centred on what Terri’s wishes would have been about treatment. Her husband and parents disagreed about this.

2 Others have suggested variations on this proposal, such as a PPP targeted at specific conditions (Ditto and Clark 2014) or a PPP for ‘meta-surrogate’ decision-making (i.e., to predict the *proxy* decision of an incapacitated surrogate; see Earp 2022).

3 Or, perhaps, the reasons, values, or evidence that the patient *endorsed*, or would have endorsed, as appropriate grounds for making treatment decisions. In fact, there are several different ways of understanding such criteria, which are meant to capture, in one way or another, what constitutes an individual’s ‘true’ reasons (etc.) for their preferences.

4 We’ve shortened to ‘P4’ rather than ‘PPPP’ to more readily distinguish the current proposal from references to the original version—the ‘PPP’—in what follows.

5 Similar opportunities exist with digital *physical* twins that simulate the body. There is already a burgeoning literature discussing ways in which autonomous persons might deliberately interact with their own digital physical twins, raising numerous ethical and philosophical questions (see Braun 2021, 2022 for overviews). What about persons who lack decision-making capacity, however, such as S in our opening example? Given the trajectory of developments in medical AI, we anticipate that it will possible in the reasonably near future for one’s *physical* digital twin to be appropriately connected to, or integrated with, one’s *psychological* digital twin (such as an advanced P4) so as to derive potentially even more precise and reliable inferences about what one would choose or want in the given circumstances, i.e., based on two different sources of information: (a) their known or extrapolated values and preferences from the P4, as applied to (b) the specifics of their current health situation as represented by their physical digital twin. This could also be an important avenue to explore for persons who may not lack capacity entirely, but who for various reasons are not able clearly to articulate their physical and psychological health needs. For further discussion of the ethics of (primarily physical) digital twins in healthcare, see Braun (2021, 2022); see also Schwartz et al. (2020). For possibilities regarding integration of physical and psychological digital twins (albeit primarily for purposes of creating a ‘personal assistant’ AI), see de Kerckhove (2021).

6 Although they float an idea that is broadly similar to the one we are exploring here, they do so in passing without much specification: “It could be argued that algorithms trained on vast amounts of individual-level data are unwieldy or even superfluous. Who needs an algorithm to suggest the same decisions people would make themselves? Such a function might become critical, however, when choices have to be made, for instance, regarding continued life support for someone who can no longer make decisions. Algorithms would not only be able to find patterns within our own past decision making but could also compare them to patterns and decisions of many other people” (1481).

7 To be clear, Lamanna and Byrne (2018) do not solely discuss population-level data sets; they, too, briefly discuss the possibility of factoring in “data provided by the patient themselves, be it implicitly through [choices] recorded on their EHR or more explicitly through social media activity” (906). We do not see an essential conflict between these different approaches or emphases. Rather, they could be seen as complementary. For example, as noted in Table 1 above, individual-level patient information could be added as a final, fine-tuned layer on top of a more general base-model LLM that was itself derived, in part, from population-level data. It may be that the greater overall volume of data afforded by such an approach (i.e., combining broad demographic correlations with person-specific information) would improve predictive accuracy in certain cases. This might be the case, for instance, in situations where the individual-level data available or authorized for a given patient is so sparse that a P4 trained exclusively on such data is unable to generate sufficiently reliable predictions. However, we acknowledge that some individuals might prefer, or only be willing to authorize, a P4 that does not include *any* generic or population-level correlations, even as part of an underlying base-model that is additionally trained on person-specific material.

8 It is important to note that, in addition to the fine-tuning mechanism we describe in this paper, there are other ways of potentially “personalizing” an LLM’s output, in the sense of adapting its output to be specific to an individual. For example, it is possible to create custom knowledge bases using writings produced by an individual, or to use custom instructions or in-context learning guided by the individual to adapt an LLM’s output to better reflect them personally. By contrast, in this paper, we envision the technical implementation of a P4 as utilizing fine-tuning as described above, due to the much larger volume of information that can be used in this method as compared to custom instructions or in-context learning, as well as the presumed ability of fine-tuned models to *infer* patient preferences (as opposed to, e.g., custom knowledge bases, which would use embeddings to reproduce existing information verbatim rather than infer preferences). Ultimately, however, it may be that a robust combination of personalization methods will be necessary to develop a successful LLM-based P4.

9 This is potentially also an objection to the use of a P4, as at least some of the information used by a P4 to infer preferences would have been produced in such a way as not to reflect the most up-to-date or accurate medical information. It is not clear that decisions based on such faulty information could reflect the patient’s real preferences. However, this is a general problem for preference prediction, and it could be addressed by supplementing (and perhaps privileging, in terms of weighting, as described above) the P4 training data with responses by the individual patient to questionnaires, surveys, or choice experiments in which accurate and up-to-date information relevant to treatment decisions had been provided to them in advance. This would partly address concerns regarding the influence of ignorance or mistaken belief about treatment options on the part of patients (i.e., it would make the preferences expressed more informed).

10 Another practical limitation of the P4 relates to introspective contributions to preference formation. Jost (2023) makes the case that introspective access to one’s affective state is a potential source of knowledge. Without access to such in-context affective experiences, an LLM may be limited in its ability to predict medically relevant preferences. However, an LLM might well be able to pick up affective contextual information through natural language processing of written text. The extent to which this is the case is an empirical question that will require further work for its resolution.

11 From another point of view, it could be argued that a study comparing conventional surrogate decision-making without a P4 (involving patient surrogates and healthcare professionals) should be compared to decision-making that additionally includes a P4. This is because the use of surrogates, PPPs, and P4s are not mutually exclusive: it is possible that each of these approaches provides valuable information to be incorporated into a larger decision-making process.
